# Incidence of Tuberculosis in Systemic Necrotizing Vasculitides: A Population-Based Study From an Intermediate-Burden Country

**DOI:** 10.3389/fmed.2020.550004

**Published:** 2020-10-22

**Authors:** Sung Soo Ahn, Minkyung Han, Juyoung Yoo, Yong-Beom Park, Inkyung Jung, Sang-Won Lee

**Affiliations:** ^1^Department of Internal Medicine, Yongin Severance Hospital, Yonsei University College of Medicine, Yongin, South Korea; ^2^Biostatistics Collaboration Unit, Department of Biomedical Systems Informatics, Yonsei University College of Medicine, Seoul, South Korea; ^3^Division of Rheumatology, Department of Internal Medicine, Yonsei University College of Medicine, Seoul, South Korea; ^4^Institute for Immunology and Immunological Diseases, Yonsei University College of Medicine, Seoul, South Korea; ^5^Division of Biostatistics, Department of Biomedical Systems Informatics, Yonsei University College of Medicine, Seoul, South Korea

**Keywords:** systemic necrotizing vasculitides, antineutrophil cytoplasmic antibody-associated vasculitis, polyarteritis nodosa, tuberculosis, risk, incidence

## Abstract

**Objective:** Tuberculosis (TB) has a significant impact on public health; however, its incidence in patients with systemic necrotizing vasculitides (SNV) remains unknown. Therefore, we evaluated the incidence of TB in patients with SNV using a nationwide claims database.

**Methods:** The Health Insurance and Review Agency database was used to identify patients diagnosed with SNV between 2010 and 2018. The standardized incidence ratio (SIR) was calculated to compared the risk of TB between patients and the general population, based on the 2016 annual national TB report. The incidence of TB after SNV diagnosis was compared by estimating age- and sex- adjusted incidence rate ratio (IRR). A time-dependent Cox regression analysis was performed to estimate factors associated with TB.

**Results:** Among the included 2,660 patients, 51 (1.9%) developed TB during the follow-up period. The risk of TB was significantly higher in patients with SNV [SIR 6.09, 95% confidence interval (CI) 4.53–8.00], both in men (SIR 5.95) and women (SIR 6.26), than in the general population; this increased risk was consistent in all disease subtypes, except eosinophilic granulomatosis with polyangiitis. Additionally, the incidence of TB was the highest in patients with SNV within the first 3 months after diagnosis (adjusted IRR: 8.90 compared to TB ≥ 12 months). In Cox regression analysis, the diagnosis of microscopic polyangiitis [hazard ratio (HR) 3.22, 95% CI 1.04–9.99], granulomatosis with polyangiitis (HR 4.63, 95% CI 1.53–14.02), and polyarteritis nodosa (HR 3.51, 95% CI 1.13–10.88) were independent factors associated with TB.

**Conclusion:** Even when considering the high incidence of TB in the geographic region, the risk of TB increased in patients with SNV, with a difference based on disease subtypes. Moreover, taking into account of the high incidence of TB in SNV, vigilant monitoring for TB is required especially during the early disease period.

## Introduction

Tuberculosis (TB) is an infectious disease caused by *Mycobacterium tuberculosis* and transmitted via the respiratory tract (1). It usually causes inflammation in the lungs of infected patients but can also affect any organ in the human body, including the lymph nodes, kidneys, central nervous system, and bones. The formation of caseating granuloma is a typical pathologic finding of TB, which consists of surrounding epithelioid macrophages and lymphocytes within a central area of necrosis (2). TB is particularly common in underdeveloped and developing countries and has a significant impact on global health. The World Health Organization reported that the incidence of TB is estimated to be approximately 10.4 million patients worldwide annually and is one of the top 10 major causes of mortality (3). Traditionally, high risk factors for developing tuberculosis are age, male sex, smoking, malnutrition, chronic diseases [chronic obstructive pulmonary disease (COPD), diabetes, chronic kidney disease (CKD)], malignancies, human immunodeficiency virus (HIV) infection, and autoimmune inflammatory rheumatic diseases [AIRDs]) (4–8). Additionally, treatment with immunosuppressive drugs that influence the immune system, such as glucocorticoids and disease-modifying anti-rheumatic drugs, is associated with the development of TB (9–11). Accordingly, research has been continuously conducted to investigate the incidence of TB in patients with AIRDs.

In general, the incidence of tuberculosis is significantly increased in patients with AIRDs than in the normal population. Rheumatoid arthritis (RA), psoriasis, and systemic lupus erythematosus (SLE) is a prototypical AIRD involving the joint, skin, and multiple organs. Previous studies have shown that the incidence of TB is elevated in patients with RA, psoriasis, and SLE (12–14). On the other hand, the occurrence of TB can also be influenced by drugs, especially anti-TNF agents, which are now widely used to treat RA, ankylosing spondylitis (AS), and psoriatic arthritis (15, 16). Furthermore, a large scale record-linkage study from the United Kingdom also suggested a heightened TB incidence in AIRDs, including RA, AS, and SLE, underscoring the importance of prudent monitoring for TB in patients with AIRDs (17).

Antineutrophil cytoplasmic antibody (ANCA)-associated vasculitis (AAV) and polyarteritis nodosa (PAN) are representative systemic necrotizing vasculitides (SNV) that manifest as the development of necrotizing inflammation within the small and medium-sized vessels (18). However, to date, literature concerning the incidence of TB in SNV is lacking. Although the development of national TB elimination programmes has led to a gradual decrease in the overall incidence and prevalence of TB, South Korea is still classified as a TB intermediate-burden country (19). Therefore, in this study, we investigated the incidence of TB and its related factors in SNV using a nationwide claims database.

## Materials and Methods

### Patient Selection and Data Extraction

We defined our patients as having SNV when they were diagnosed as AAV or PAN at a general or tertiary care hospital and were prescribed with glucocorticoids (methylprednisolone, hydrocortisone, prednisone, prednisolone, triamcinolone, budesonide, betamethasone, dexamethasone, or deflazacort) during the follow-up. To select SNV cases, the corresponding International Classification of Diseases (ICD)-10 codes for microscopic polyangiitis (MPA), granulomatosis with polyangiitis (GPA), eosinophilic granulomatosis with polyangiitis (EGPA), and PAN (M31.7, M31.3, M30.1, and M30.0, respectively) were used (20). Concerning the medications used by patients after the diagnosis of SNV and prior to the incidence of TB, the usage of glucocorticoids, cyclophosphamide, rituximab, azathioprine, and methotrexate was determined. Methotrexate dosage that was above the standard dose prescribed for AAV (>25 mg/week) was excluded.

To acquire data of patients with SNV, we searched the Health Insurance and Review Agency (HIRA) data from January 2008 to December 2018. HIRA database is a nationwide claims database that contains information on the use of medical services, including hospital visit (including hospitalization and ambulatory care) and drug prescription for the majority of health care users (>50 million people in South Korea) included in the national health insurance service. In detail, HIRA database contains information regarding age, sex, insurance type, diagnosis, comorbidity using ICD-10 codes, prescriptions of medications, and the utilization of healthcare related procedures of individuals (21).

In the present study, the first date of registration of the corresponding ICD-10 codes in the database was regarded as the date of SNV diagnosis (index date). A 2-year washout period was applied to exclude prevalent SNV cases. The follow-up duration of patients was calculated from the index date of SNV until the occurrence of TB or the last follow-up. This study was approved by the institutional review board of the hospital and was performed according to the principles set by the Declaration of Helsinki. The requirement to obtain informed consent was waived, as this study was performed retrospectively (4-2019-0177).

### Definition of TB in Patients With SNV and Investigation of Comorbidities

Patients with SNV who were assigned the ICD-10 codes of TB (A15-19) after SNV diagnosis and were prescribed with at least two of the first-line drugs for TB (isoniazid, rifampin/rifampicin, pyrazinamide, ethambutol) at the date of TB diagnosis were defined as incident TB cases (22–24). The number of expected cases with TB was calculated by multiplying the person-years of patients with AAV and age- and sex-specific TB incidence rate based on the 2016 annual national TB report (25).

The investigated comorbidities associated with TB included the presence of hypertension (ICD-10 code I10-15), diabetes mellitus (E10-14), CKD (N18), COPD (J43-44), HIV infection (B20-24), and silicosis (J62), which were searched for using the ICD-10 codes of patients within 1 year of the index date of SNV.

### Statistical Analysis

Continuous and categorical variables were presented as mean ± standard deviation and frequencies (percentages). The Student's *t*-test was used to compare continuous variables, and the chi-square or Fisher's exact tests were used to compare categorical variables, as appropriate. Patients were divided into 10-year intervals by age, and standardized incidence ratios (SIRs) of the corresponding interval were calculated to compare the incidence of TB between patients with SNV and in the general population. SIRs were estimated by dividing the number of observed cases by that of expected cases, and the 95% confidence interval (CI) was estimated using Poisson distribution. To compare the incidence of TB after SNV diagnosis, the incidence rate per 1,000 person-years and an age- and sex- adjusted incidence rate ratio (IRR) were estimated by dividing the intervals into four groups (TB <3 months, 3 ≤ TB <6 months, 6 ≤ TB <12 months, and TB ≥ 12 months). The Kaplan-Meier method and log-rank test were used to the estimate and compare cumulative incidence rates of TB according to disease subtypes.

To investigate clinical factors associated with TB in patients with SNV, a time-dependent Cox regression analysis was performed. Age, sex, type of diagnosis, insurance status, and comorbidities were included as time-fixed variables. In addition, in order to avoid length bias, the administration of immunosuppressive agents of glucocorticoid usage ≥12 months, cyclophosphamide, azathioprine, methotrexate, and rituximab was included as time-dependent variables to calculate the hazard ratios (HRs) of TB occurrence. In all statistical analyses, *P*-value < 0.05 was considered significant, and SAS 9.4 Enterprise Guide (SAS Institute, Inc.) and R 3.6.1 (R Foundation for Statistical Computing, Vienna, Austria) were used for all statistical analyses.

## Results

### Clinical Characteristics of SNV Patients With TB and Without TB

**Figure 1:**
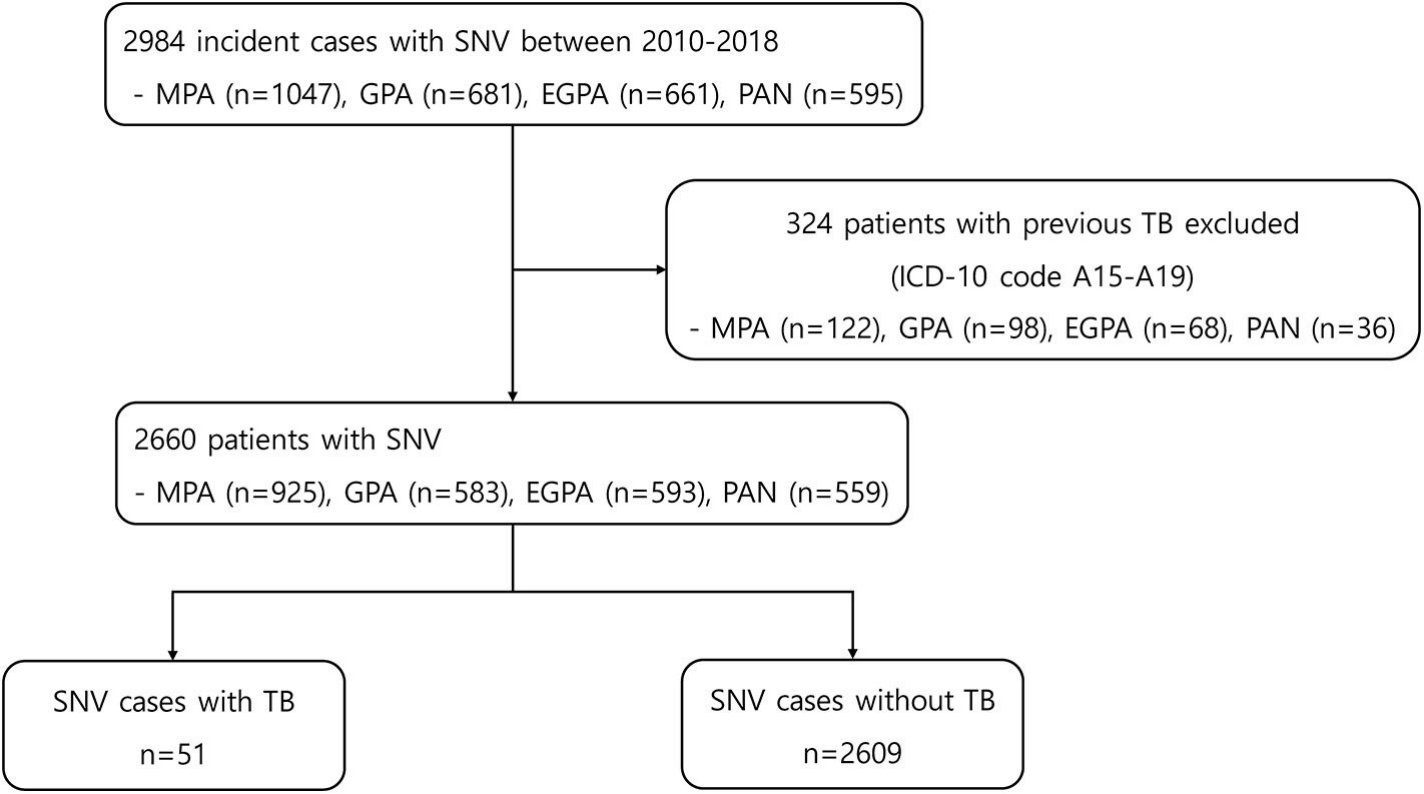
Flowchart for patient selection and data extraction. SNV, systemic necrotizing vasculitides, MPA, microscopic polyangiitis; GPA, granulomatosis with polyangiitis; EGPA, eosinophilic granulomatosis with polyangiitis; PAN, polyarteritis nodosa; TB, tuberculosis; ICD, international classification of diseases.

At baseline, 2,984 incident cases of SNV were searched. Among them, 324 patients were excluded because of a previous history of TB (Figure 1). The mean age of the remaining 2,660 patients was 57.5 years, and 54.8% were women. MPA was the most common diagnosis (34.8%) within SNV, followed by EGPA, GPA, and PAN. During the mean follow-up period of 3.3 years and 8,796.85 person-years, 51 patients (1.9%) developed TB and 2,609 (98.1%) did not. The mean follow-up duration was 1.4 years (median 0.5 years and 72.14 person-years) for patients with TB and 3.3 years (median 2.8 years and 8,724.71 person-years) for those without TB (*P* < 0.001). Comparison of baseline characteristics between the two groups showed no significant difference in age, sex, insurance status, and the presence of comorbidities. However, the proportion of patients with EGPA was lower in patients with TB [7.8% in the TB (+) group and 22.6% in the TB (–) group], and the proportion of patients prescribed glucocorticoids for ≥12 months and azathioprine was significantly lower in the TB group (*P* < 0.001 and *P* = 0.013) (Table 1).

**Table 1:** Clinical characteristics of patients with SNV at initial diagnosis.

	**Total patients *n* = 2,660**	**Patient with TB *n* = 51**	**Patient without TB *n* = 2,609**	***P*-value**
Age, years	57.5 ± 16.9	59.1 ± 18.1	57.5 ± 16.8	0.502
Sex, *n* (%)				0.208
Male	1,203 (45.2)	28 (54.9)	1,175 (45.0)	
Female	1,457 (54.8)	23 (45.1)	1,434 (55.0)	
Diagnosis [ICD-10 code], *n* (%)				0.037
MPA	925 (34.8)	17 (33.3)	908 (34.8)	
GPA	583 (21.9)	17 (33.3)	566 (21.7)	
EGPA	593 (22.3)	4 (7.8)	589 (22.6)	
PAN	559 (21.0)	13 (25.5)	546 (20.9)	
Follow-up duration, years	3.3 ± 2.7	1.4 ± 2.0	3.3 ± 2.7	<0.001
Type of TB, *n* (%)				
Pulmonary TB [A15, 16, 19]	43 (84.3)	43 (84.3)		
Extrapulmonary TB [A17-18]	8 (15.7)	8 (15.7)		
Insurance status, *n* (%)				1.000
National Health Insurance	2,550 (95.9)	49 (96.1)	2,501 (95.9)	
Medical Aid	110 (4.1)	2 (3.9)	108 (4.1)	
**COMORBIDITIES [ICD-10 CODE]**, ***n*** **(%)**				
Hypertension [I10-15]	1,255 (47.2)	27 (52.9)	1,228 (47.1)	0.490
Diabetes mellitus [E10-14]	899 (33.8)	19 (37.3)	880 (33.7)	0.706
Chronic kidney disease [N18]	349 (13.1)	11 (21.6)	338 (13.0)	0.111
Chronic obstructive pulmonary disease [J43-44]	427 (16.1)	9 (17.7)	418 (16.0)	0.904
HIV infection [B20-24]	0 (0.0)	0 (0.0)	0 (0.0)	n/a
Silicosis [J62]	0 (0.0)	0 (0.0)	0 (0.0)	n/a
**IMMUNOSUPPRESSIVE AGENTS USAGE**, ***n*** **(%)**				
Glucocorticoid usage ≥ 12 month	1,246 (46.8)	10 (19.6)	1,236 (47.4)	<0.001
Cyclophosphamide	1,153 (43.4)	18 (35.3)	1,135 (43.5)	0.304
Azathioprine	992 (37.3)	10 (19.6)	982 (37.6)	0.013
Methotrexate	433 (16.3)	7 (13.7)	426 (16.3)	0.759
Rituximab	258 (9.7)	3 (5.9)	255 (9.8)	0.476

Values are expressed as mean (standard deviation) or in number (percentages).

*SNV, systemic necrotizing vasculitides; TB, tuberculosis; ICD, international classification of diseases; MPA, microscopic polyangiitis; GPA, granulomatosis with polyangiitis; EGPA, eosinophilic granulomatosis with polyangiitis; PAN, polyarteritis nodosa; HIV, human immunodeficiency virus*.

### Calculation of TB Risk in Patients With SNV

**Figure 2:**
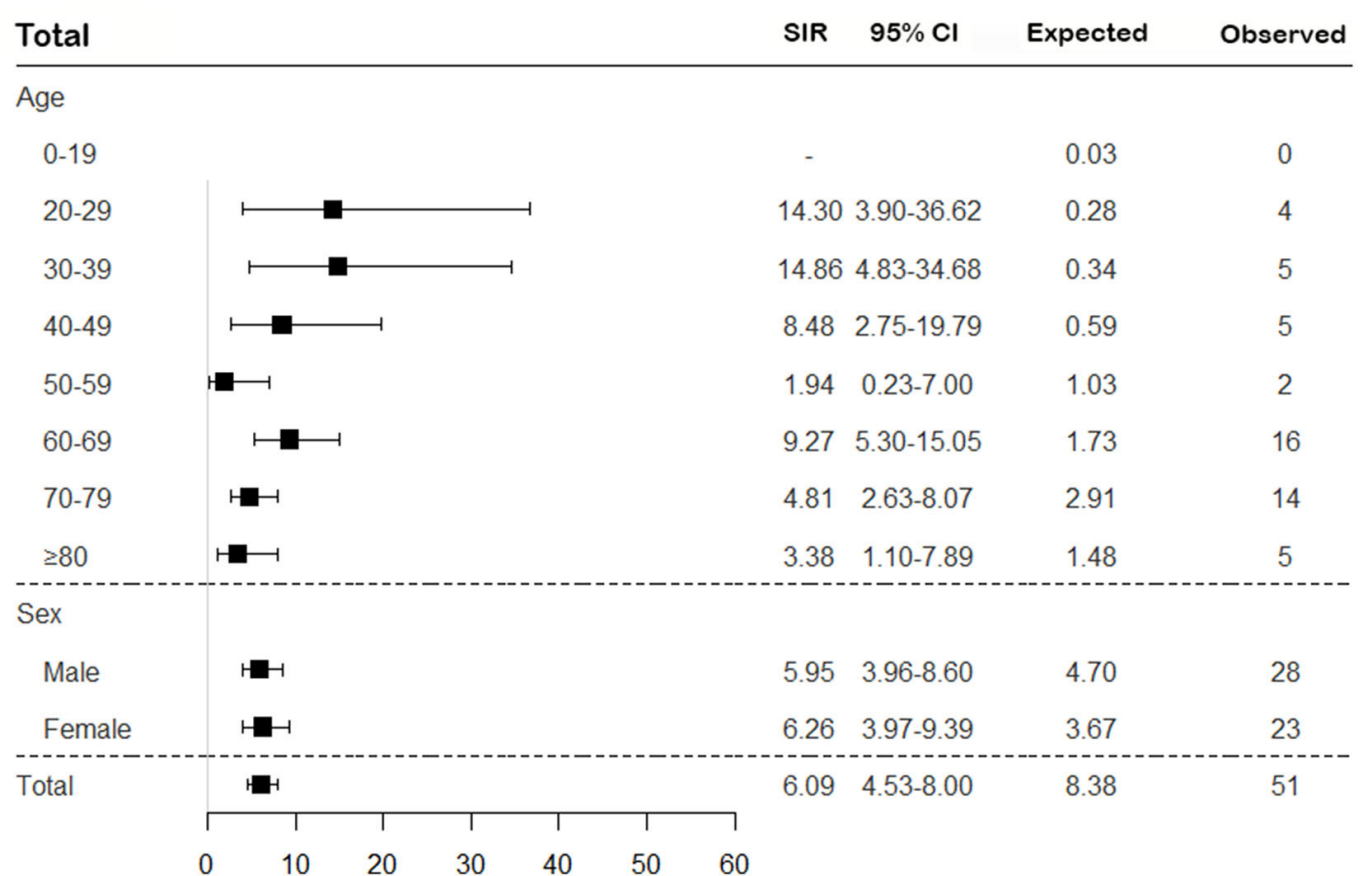
Assessment of the risk of TB in patients with SNV according to age and sex. The overall SIR of TB was estimated in patients with SNV compared to the general population. TB, tuberculosis; SNV, systemic necrotizing vasculitides; SIR, standardized incidence ratio; CI, confidence interval.

**Figure 3:**
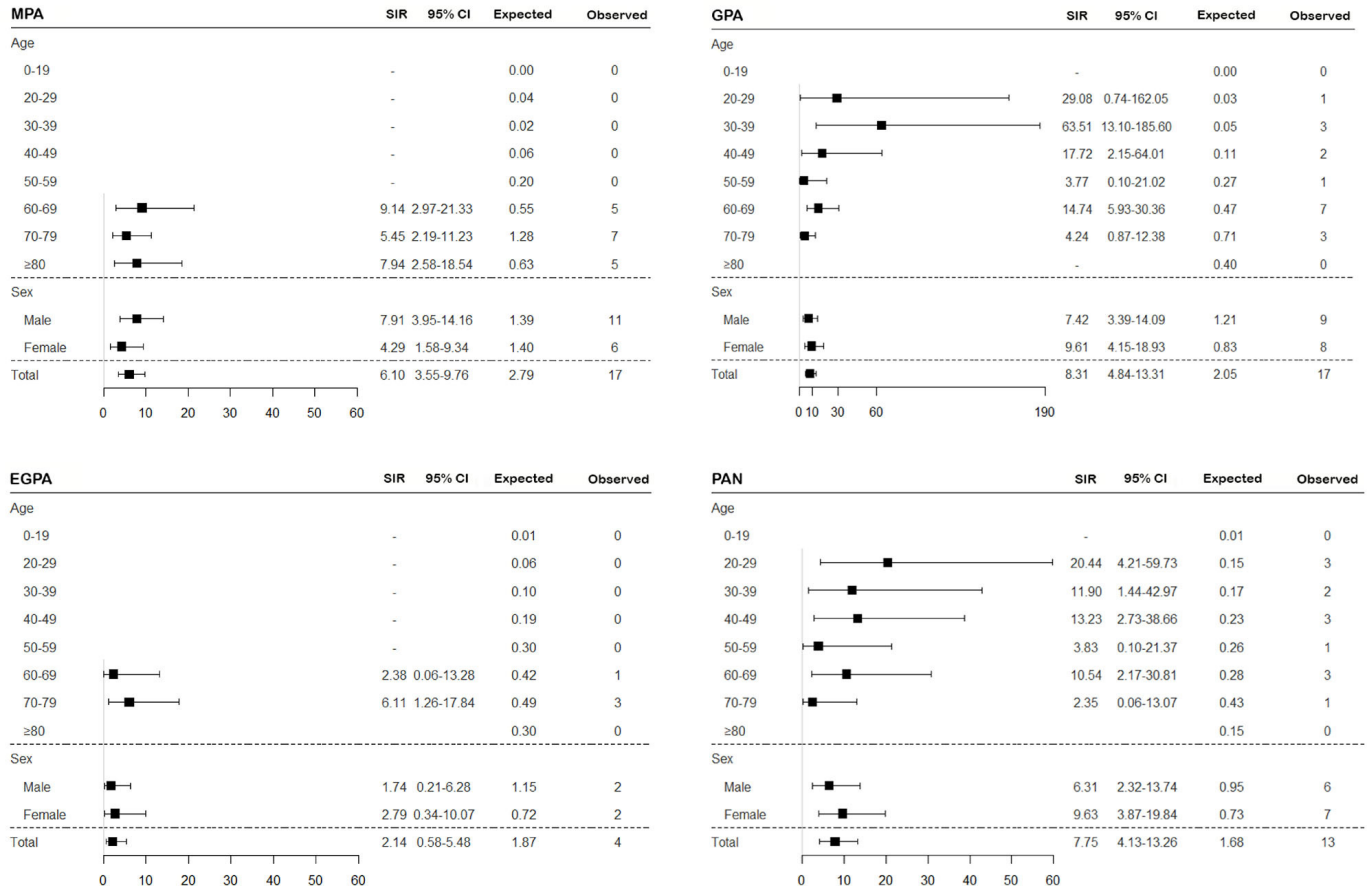
Assessment of the risk of TB in SNV subtypes according to age and sex. The SIR of TB was estimated in patients with MPA, GPA, EGPA, and PAN compared to the general population. TB, tuberculosis; SNV, systemic necrotizing vasculitides; SIR, standardized incidence ratio; MPA, microscopic polyangiitis; GPA, granulomatosis with polyangiitis; EGPA, eosinophilic granulomatosis with polyangiitis; PAN, polyarteritis nodosa; CI, confidence interval.

To assess the risk of TB in patients with SNV, the incidence of TB was calculated, and SIRs were obtained by comparing to the general population based on the 2016 annual national TB report. Overall, the risk of TB in SNV was higher in patients with SNV than in the general population (SIR 6.09, 95% CI 4.53–8.00) and was consistently higher for all subtypes of SNV, except EGPA (SIR 2.14, 95% CI 0.58–5.48) (Figures 2, 3). On comparing the risk of TB according to 10-year age intervals, the risk of TB was significantly higher in all age groups of SNV, except for those aged 0–19 and 50–59 years. In particular, SIRs of TB were the highest in patients aged 20–29 and 30–39 years (SIR 14.30, 95% CI 3.90–36.62; SIR 14.86, 95% CI 4.83–34.68). The risk of TB was equally high in patients with SNV regardless of sex (SIR 5.95, 95% CI 3.96–8.60 in men and SIR 6.26, 95% CI 3.97–9.39 in women). The risk of TB was not elevated in either men or women with EGPA (Figures 2, 3).

**Figure 4:**
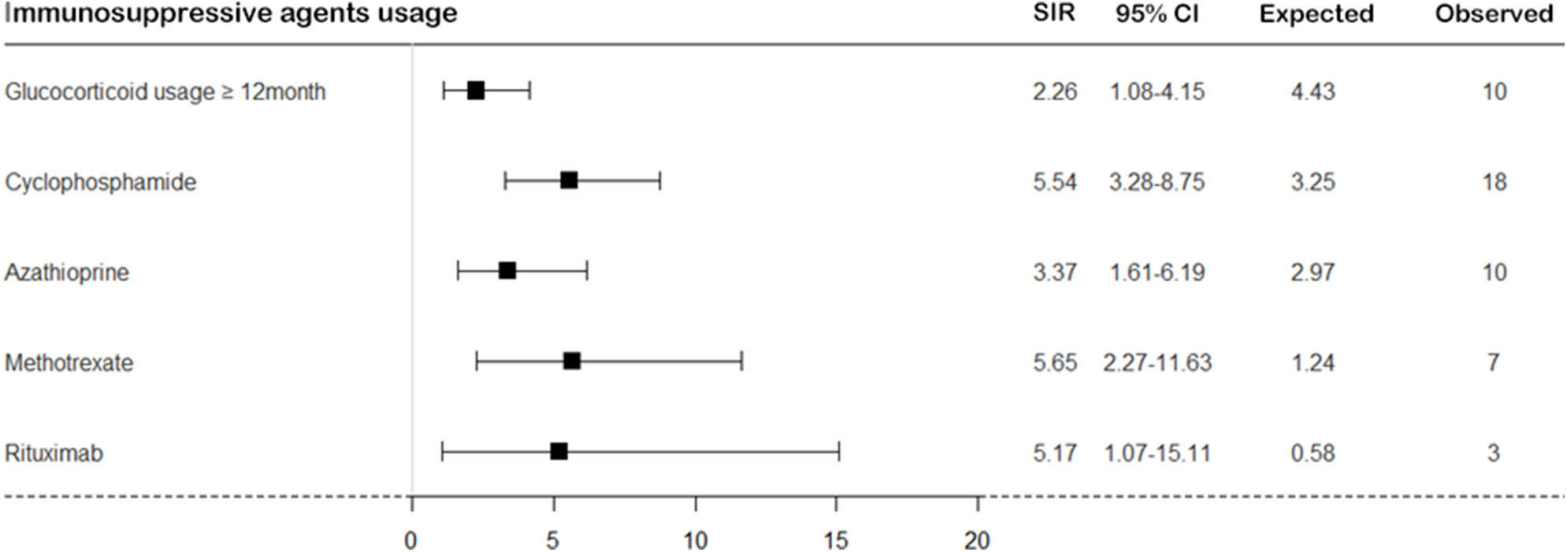
The risk of TB in patients with SNV according to immunosuppressive agent usage. The risk of TB was calculated in patients with SNV compared to the general population according to immunosuppressive agent usage. TB, tuberculosis; SNV, systemic necrotizing vasculitides; SIR, standardized incidence ratio; CI, confidence interval.

**Figure 5:**
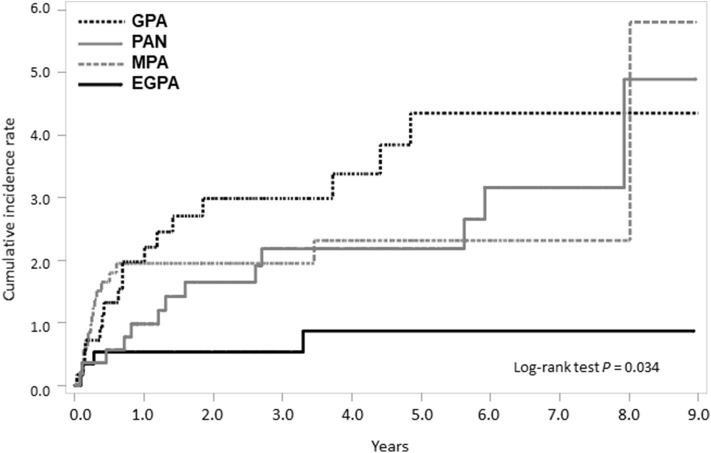
Comparison of cumulative TB incidence rate among SNV subtypes. TB, tuberculosis; SNV, systemic necrotizing vasculitides; GPA, granulomatosis with polyangiitis; PAN, polyarteritis nodosa. MPA, microscopic polyangiitis; EGPA, eosinophilic granulomatosis with polyangiitis.

On calculating the risk of TB according to medication usage, the risk of TB was significantly higher in patients with SNV treated with immunosuppressive agents than in the general population (Figure 4). When we divided patients as per the disease subtypes and compared the cumulative incidence rate of TB, a significant difference was found in the occurrence of TB; the cumulative incidence of TB was the lowest in patients with EGPA (log-rank test *P* = 0.034) (Figure 5).

### Risk of TB in Patients With SNV After Disease Diagnosis

Next, we evaluated whether the risk of TB differed in patients with SNV according to the period after diagnosis. When the incidence of TB was calculated in the patients after disease diagnosis, the incidence rate/1,000 person-years was especially higher in TB <3 months (26.07/1,000), followed by 3 months ≤ TB <6 months (18.06/1,000), 6 months ≤ TB <12 months (6.94/1,000), and TB ≥ 12 months (2.72/1,000). Furthermore, IRR of TB in SNV within 3 months of diagnosis was significantly higher than TB ≥ 12 months in the unadjusted (IRR 9.59 compared to TB ≥ 12 months, 95% CI 4.89–18.81) and in age- and sex-adjusted (IRR 8.90 compared to TB ≥ 12 months, 95% CI 4.52–17.53) analyses (Table 2). In all disease types, the incidence rate of TB was higher during the early period after diagnosis (Table 3).

**Table 2:** Incidence rate ratio of TB in patients with SNV according to the interval after diagnosis.

	**Total**				
**After diagnosis**	**# of Events**	**PY**	**IR/1,000 PY (95% CI)**	**Crude IRR (95% CI)**	**Adjusted IRR (95% CI)^**¶**^**
TB <3 months	16	613.83	26.07 (15.29–41.00)	9.59 (4.89–18.81)	8.90 (4.52–17.53)
3 months ≤ TB <6months	10	553.74	18.06 (9.05–31.67)	6.63 (3.06–14.37)	6.27 (2.89–13.61)
6 months ≤ TB <12 months	7	1,008.50	6.94 (2.98–13.42)	2.55 (1.07–6.11)	2.44 (1.02–5.84)
TB ≥12 months	18	6,620.78	2.72 (1.65–4.17)	1.00 (Ref)	1.00 (Ref)

¶ IRR was calculated by adjusting for age and sex.

*TB, tuberculosis; SNV, systemic necrotizing vasculitides; PY, person-years; IR/1,000 PY, incidence rate/1,000 person-years; CI, confidence interval; IRR, incidence rate ratio*.

**Table 3:** Risk of TB in subtypes of SNV after diagnosis.

	**MPA**			**GPA**			**EGPA**			**PAN**		
**After diagnosis**	**# of Events**	**PY**	**IR/1,000 PY (95% CI)**	**# of Events**	**PY**	**IR/1,000 PY (95% CI)**	**# of Events**	**PY**	**IR/1,000 PY (95% CI)**	**# of Events**	**PY**	**IR/1,000 PY (95% CI)**
TB <3 months	8	205.34	38.96 (17.80–72.51)	4	135.84	29.45 (9.14–68.40)	2	139.93	14.29 (2.38–44.11)	2	132.72	15.07 (2.51–46.50)
3 months ≤ TB <6 months	5	173.99	28.74 (10.30–61.76)	3	122.76	24.44 (6.08–63.35)	1	131.98	7.58 (4.32–33.33)	1	125.01	8.00 (0.46–35.19)
6 months ≤ TB <12 months	2	304.36	6.57 (1.09–20.28)	3	219.67	13.66 (3.40–35.40)	0	248.31	–	2	236.16	8.47 (1.41–26.13)
TB ≥12 months	2	1,599.62	1.25 (0.21–3.86)	7	1,479.66	4.73 (2.03–9.15)	1	1,646.01	0.61 (0.03–2.67)	8	1,895.49	4.22 (1.93–7.86)

*TB, tuberculosis; SNV, systemic necrotizing vasculitides; MPA, microscopic polyangiitis; GPA, granulomatosis with polyangiitis; EGPA, eosinophilic granulomatosis with polyangiitis; PAN, polyarteritis nodosa; PY, person-years; IR/1,000 PY, incidence rate/1,000 person-years; CI, confidence interval*.

### Factors Associated With the Risk of TB in Patients With SNV

In the unadjusted Cox regression analysis, the diagnoses of MPA (HR 3.45, 95% CI 1.16–10.26), GPA (HR 4.58, 95% CI 1.54–13.62), PAN (HR 3.20, 95% CI 1.04–9.81), and the comorbidity of CKD (HR 2.20, 95% CI 1.13–4.29) were significant factors associated with TB. However, in the adjusted analysis, only the diagnoses of MPA (HR 3.22, 95% CI 1.04–9.99), GPA (HR 4.63, 95% CI 1.53–14.02), and PAN (HR 3.51, 95% CI 1.13–10.88) were significantly associated with the increased risk of TB (Table 4).

**Table 4:** Factors associated with the occurrence of TB during follow-up.

	**Crude hazard ratio**		**Adjusted hazard ratio**	
	**Hazard ratio (95% CI)**	***P*-value**	**Hazard ratio (95% CI)**	***P*-value**
Age^‡^	1.02 (0.99–1.03)	0.087	1.01 (0.99–1.03)	0.412
**SEX**^**‡**^				
Male	1.55 (0.89–2.69)	0.119	1.48 (0.85–2.60)	0.168
Female	1.00 (Ref)		1.00 (Ref)	
**DIAGNOSIS**^**‡**^				
MPA	3.45 (1.16–10.26)	0.026	3.22 (1.04–9.99)	0.043
GPA	4.58 (1.54–13.62)	0.006	4.63 (1.53–14.02)	0.007
EGPA	1.00 (Ref)		1.00 (Ref)	
PAN	3.20 (1.04–9.81)	0.042	3.51 (1.13–10.88)	0.030
**INSURANCE STATUS**^**‡**^				
National health insurance	1.00 (Ref)		1.00 (Ref)	
Medical aid	0.95 (0.23–3.91)	0.946	1.06 (0.29–3.87)	0.926
**COMORBIDITIES**^**‡**^				
Hypertension	1.49 (0.86–2.59)	0.156	1.11 (0.58–2.11)	0.751
Diabetes mellitus	1.31 (0.74–2.32)	0.346	1.06 (0.57–1.96)	0.852
Chronic kidney disease	2.20 (1.13–4.29)	0.021	1.77 (0.83–3.75)	0.138
Chronic obstructive pulmonaryDisease	1.23 (0.60–2.53)	0.568	1.45 (0.68–3.08)	0.336
**IMMUNOSUPPRESSIVE AGENTS USAGE**^**†**^				
Glucocorticoid usage ≥ 12 month	0.86 (0.30–2.50)	0.787	1.10 (0.36–3.30)	0.870
Cyclophosphamide	0.87 (0.49–1.54)	0.628	0.76 (0.41–1.40)	0.381
Azathioprine	0.64 (0.32–1.31)	0.222	0.62 (0.30–1.28)	0.199
Methotrexate	1.11 (0.50–2.44)	0.799	1.01 (0.44–2.33)	0.974
Rituximab	1.12 (0.37–3.35)	0.840	0.91 (0.29–2.82)	0.871

‡ Age, sex, type of diagnosis, insurance status, and comorbidities were included as time-fixed variables.

Glucocorticoid usage ≥12 months, cyclophosphamide, azathioprine, methotrexate, and rituximab usage were included as time-dependent variables.

*TB, tuberculosis; CI, confidence interval; MPA, microscopic polyangiitis; GPA, granulomatosis with polyangiitis; EGPA, eosinophilic granulomatosis with polyangiitis; PAN, polyarteritis nodosa*.

## Discussion

Accumulating evidence now indicate that the risk of TB is elevated in patients with AIRDs; however, the incidence of TB in patients with SNV remains unclear. In this study, we evaluated the incidence of TB in patients with SNV using the National Health Insurance Claims Database. Our results revealed that 1.9% (*n* = 51) of the patients developed TB during follow-up. Remarkably, the risk of TB was over six times higher in patients with SNV than in the general population, consistent with findings of previous studies that demonstrated increased risks of TB in patients with AIRDs. Moreover, the risk of TB was the most pronounced within the first 3 months after SNV diagnosis. Among disease subtypes, the risk of TB was elevated in patients with MPA, GPA, and PAN, and the highest risk was observed in GPA compared to the general population. The strength of this study is that, to the best of our knowledge, this is the first to evaluate the incidence of TB in patients with SNV using a nationwide database.

Two possible hypotheses could be drawn regarding the increased risk of TB in SNV found in this study. First, in the pathogenesis, of SNV, abnormalities of the innate and adaptive immune systems are responsible for the development of chronic inflammation and perpetuation of the vicious cycle (26). Accumulating evidences now suggest that a defect in adaptive immune response, especially T cells, is important in the pathogenesis of SNV (27). Similarly, hampered clearance of TB by the immune system is important in the development of TB, and the generation of effective CD4+ T-cell response is crucial in the host defense against TB (28). Therefore, the incidence of TB may be higher in patients with SNV due to functional impairment of host immunity. Second, continuous administration of glucocorticoids and immunosuppressive drugs could also be attributable to the development of TB. Glucocorticoids and immunosuppressive drugs have been demonstrated to exhibit broad effects on suppressing the immune response, as well as inhibiting T-cell activation and differentiation (29–32). Accordingly, the use of glucocorticoids and immunosuppressive drug may be associated with the risk of TB.

Although the incidence of TB in South Korea is decreasing, TB remains to be one of the most important public health issues in this endemic region, owing to the high infectivity and difficulty to effectively treat at-risk populations (33). According to the 2016 World Health Organization TB estimates, the incidence of TB is 77 cases per 100,000 persons in South Korea, which was higher than in the United States or European countries (2.9 cases per 100,000 persons in United States; 31.6 cases per 100,000 persons in Europe) (3, 34). However, even when considering the high incidence of TB in the geographic region, our findings have revealed that SIR for TB was over six times (SIR 6.09) in patients with SNV than in the general population, and the increased risk of TB was identical regardless of sex. Moreover, the risk of TB was consistently elevated in all age groups, only except in those aged <20 and 50–59 years. Although the incidence of tuberculosis increases with age in the general population according to the 2016 annual national TB report, SIRs of TB were higher in younger patients with SNV (25). These findings imply that careful monitoring is necessary for these patients at all age groups for the development of TB. However, given that older patients are more likely to drop-out as a result of higher mortality, it is unclear whether this observation is related to higher TB susceptibility in younger SNV patients or is a consequence of higher mortality rates and drop-out of older SNV patients, which should be verified through future investigations.

The incidence of TB was particularly higher within the first 3 months of SNV diagnosis. Because the normal immune response is compromised in patients with SNV, patients may be more subject to TB during the early phase of SNV diagnosis, in which perturbation of the immunity is the most prominent. Furthermore, immunosuppressants are most actively used during the early stages to achieve disease remission; thus, the incidence of TB could increase during the early phase of the disease because of the higher requirement of immunosuppressive drugs. However, although patients with SNV treated with immunosuppressive agents exhibited an equally increased risk of TB compared to the general population, the association between immunosuppressive agents and the occurrence of TB in SNV was not found in the Cox regression analysis, suggesting that drugs used to treat SNV did not have a significant impact in the development of TB by themselves. This discrepancy could be attributed to the largely variable incidence of TB in chronic inflammatory diseases (35). The difference in the geographies, ethnicity, and diseases investigated might have also influenced our results. Moreover, the risk of TB in those undergoing immunosuppressive treatment may be increased in a dose-dependent manner, and the dose of drugs used were not sufficiently high to provoke TB (36). However, because we could not evaluate detailed drug dosages for the patients, further studies are required to better understand the relationship between immunosuppressants and the risk of TB in SNV.

In the subgroup analysis according to disease subtypes of SNV, the risk of TB was significantly higher in all subtypes than in the general population, except for EGPA. Among the disease subtypes, the risk of TB was the highest in patients with GPA. Furthermore, in Cox regression analysis, the HR of GPA was the highest within SNV subtypes. Although the exact cause of this disease specific-predisposition is unclear, it could be related to GPA being defined as necrotizing granulomatous inflammation most commonly involving the upper and lower respiratory tracts (37). Radiographic findings of nodular, cavitary lesions, and consolidations in the lung parenchyma are common in patients with GPA (38). Considering that chronic inflammation in the respiratory tracts is associated with TB (39), it could be hypothesized that the risk of TB is elevated in patients with SNV, especially GPA. Nevertheless, as asthma and pulmonary involvement are also often present in EGPA, additional investigations are necessary to elucidate why patients with EGPA are less susceptible to TB. Meanwhile, as imaging findings of GPA can be difficult to discriminate from those of TB, a pathological confirmation to exclude the possibility of TB is essential if a clinical diagnosis of TB is suspected.

In the present study, the number of patients with extrapulmonary TB was 8 (15.7%), which seems to be numerically similar to the proportion of extrapulmonary TB arising in immunocompetent subjects and is contradictory to the knowledge that patients under TNF-α inhibitors are more prone to extrapulmonary TB (40). In addition, even though the number of incident TB cases were not large, a recent publication by Chung et al. reported that a proportion of extrapulmonary TB in patients with rheumatoid arthritis (RA) was 38.5% (41), and a study by Vuorela et al. has shown that ~23.9% of patients with rheumatic diseases are affected with extrapulmonary TB (42). Several factors could be accounted for the discrepant results between the previous studies and our study. Even though TNF-α inhibitors increases the risk of extrapulmonary TB, TNF-α blocking agents are not generally recommended for the treatment of SNV (43). Therefore, the high incidence of extrapulmonary TB in a RA cohort could be explained by the frequent use of TNF-α inhibition for RA treatment. In addition, it is also possible that a difference between AIRDs could be present concerning the development of pulmonary or extrapulmonary TB, especially in SNV. Finally, because our study was conducted only by using the ICD-10 codes of the patients, the clinical details may not be sufficiently reflected in classifying as pulmonary and extrapulmonary TB.

Several limitations are present in this study. First, clinical details regarding SNV, including disease severity, patterns of organ involvement, and laboratory data, were not available. Second, because there are no established criteria to identify SNV and TB using administrative data, differences in the definition used to define SNV and TB might have influenced patient selection. In particular, identification of TB cases by using the ICD-10 codes and 1st line medications should be further verified in population based studies. Third, the clinical outcome of patients affected with TB and the effect of TB medications may not be fully addressed through the HIRA data. Fourth, it remains unclear whether patients were routinely screened and treated for latent TB before immunosuppressive treatment and pre-emptive therapy for high-risk individuals (e.g., those with latent TB or elderly patients) are required to reduce the risk of TB.

In conclusion, patients with SNV were prone to TB than the general population. The risk of TB was higher within the first 3 months after SNV diagnosis, and it was highest in those with GPA among the disease subtypes. Our findings emphasize that vigilant monitoring for the occurrence of TB is required in the management of patients with SNV, especially during the early disease period.

## Data Availability Statement

The raw data supporting the conclusions of this article will be made available by the authors, without undue reservation.

## Ethics Statement

The studies involving human participants were reviewed and approved by Institutional Review Board of Yonsei University (IRB approval number 4-2019-0177). Written informed consent from the participants' legal guardian/next of kin was not required to participate in this study in accordance with the national legislation and the institutional requirements.

## Author Contributions

SA designed the report and wrote the paper. MH and JY participated in data acquisition and interpretation. Y-BP, IJ, and S-WL drafted and revised the manuscript. S-WL designed the concept and approved the final paper. All authors have taken care to ensure the integrity of this work, and the final manuscript has been seen and approved by all authors. All authors contributed to the article and approved the submitted version.

## Conflict of Interest

The authors declare that the research was conducted in the absence of any commercial or financial relationships that could be construed as a potential conflict of interest.
